# How COVID-19 lockdown and reopening affected daily steps: evidence based on 164,630 person-days of prospectively collected data from Shanghai, China

**DOI:** 10.1186/s12966-021-01106-x

**Published:** 2021-03-17

**Authors:** Ding Ding, Minna Cheng, Borja del Pozo Cruz, Tao Lin, Shuangyuan Sun, Li Zhang, Qinping Yang, Zhicong Ma, Jing Wang, Yingnan Jia, Yan Shi

**Affiliations:** 1 Prevention Research Collaboration, Sydney School of Public Health, Faculty of Medicine and Health, The University of Sydney, Camperdown, New South Wales Australia; 2 Charles Perkins Centre, The University of Sydney, Camperdown, New South Wales Australia; 3 Shanghai Municipal Center for Disease Control and Prevention, 1380 West Zhongshan Road, Shanghai, 200336 China; 4 Centre for Active and Healthy Ageing, Department of Sports Science and Clinical Biomechanics, University of Southern Denmark, Campusvej 55, 5230 Odense, Denmark; 5 Shanghai Pudong New Area Center for Disease Control and Prevention, Shanghai, 200136 China; 6 School of Public Health, Key Lab of Public Health Safety of the Ministry of Education, Fudan University, 130 Dongan Road, Shanghai, 200032 China; 7 Health Communication Institute, Fudan University, Shanghai, 200032 China; 8 National Clinical Research Center for Aging and Medicine, Huashan Hospital, Fudan University, Shanghai, 200040 China

## Abstract

**Background:**

COVID-19 lockdowns may lead to physical inactivity, a major risk factor for non-communicable diseases. This study aims to determine: 1) the trajectory in daily step counts before, during and after the lockdown in China, and 2) the characteristics associated with the trajectories.

**Methods:**

From December 2019 to July 2020, smartphone-based step counts were continuously collected in 815 Chinese adults residing in Shanghai over 202 days across three phases: before, during, and after the lockdown. Participant characteristics were reported, and height, weight and body composition measured before the lockdown. A ‘sharp’ regression discontinuity design with cluster robust standard errors was used to test the effect of the lockdown and reopening on daily steps and a linear mixed model was used to examine the characteristics associated with trajectories during the observed period.

**Results:**

Based on 164,630 person-days of data, we found a sharp decline in daily step counts upon the lockdown (24/01/2020) by an average of 3796 (SE = 88) steps, followed by a significant trend of increase by 34 steps/day (SE = 2.5; *p* < .001) until the end of the lockdown (22/03/2020). This increasing trend continued into the reopening phase at a slower rate of 5 steps per day (SE = 2.3; *p* = 0.029). Those who were older, married, university educated, insufficiently active, had an ‘at risk’ body composition, and those in the control group, were slower at recovering step counts during the lockdown, and those who were older, married, without university education and with an ‘at risk’ body composition recovered step counts at a slower pace after the reopening.

**Conclusions:**

Despite later increases in step counts, COVID-19 lockdown led to a sustained period of reduced physical activity, which may have adverse health implications. Governments and health professionals around the world should continue to encourage and facilitate physical activity during the pandemic.

**Supplementary Information:**

The online version contains supplementary material available at 10.1186/s12966-021-01106-x.

## Introduction

First reported in Wuhan, China, in December 2019, Coronavirus disease-2019 (COVID-19) has quickly spread around the world, causing colossal health, social and economic damage. On March 11, 2020, the World Health Organization (WHO) declared COVID-19 a pandemic, [1] and by 30 Sep 2020, more than 33 million confirmed cases have been reported in more than 200 countries, resulting in over a million deaths worldwide [2]. To contain the spread of the virus, most governments around the world have adopted lockdowns and physical distancing measures. Such restrictions, although necessary for infection control, impose potential consequences in other areas of public health, such as cancer screening, [3] domestic violence, [4] mental health problems [5] and chronic disease management [6].

Of all the risk factors potentially affected by COVID-19 lockdowns, physical inactivity is among the most studied. Mounting evidence supports the role physical activity plays in the prevention and management of chronic illness, such as coronary heart disease, type 2 diabetes and some cancers [7, 8]. A recent study based on the large UK Biobank cohort also found that regular physical activity may protect against COVID-19 hospitalisation, an indicator of severe symptoms [9]. Since initial lockdown restrictions have been implemented, several online surveys have been conducted to examine how physical activity levels changed as a result of the lockdown. A multi-country survey found a significant reduction in all intensity levels of physical activity during home confinement [10]. Similar reductions in physical activity have been confirmed by studies in Spain, [11] Australia [12] and Chile, [13] while a study in Belgium found an increase in exercise frequency [14]. While these studies provide useful insights into the effects of COVID-19 lockdowns on physical activity, they are subject to several limitations: first, nearly all studies are based on cross-sectional or retrospective data collections, without having a ‘real’ baseline prior to the lockdown; second, these studies are based on self-reported physical activity only, which is subject to measurement bias; third, except for a few studies that examined sub-group differences, [15, 16] most studies only reported the overall change in physical activity without investigating the characteristics associated with change in physical activity; fourth, nearly all studies examined the effects of the lockdown only without investigating the effects of reopening. The only way to avoid these major limitations is analysis of retrospectively obtained device data, which was done by one study to our knowledge [17]. This study found world-wide declines in step counts following the COVID-19 lockdown. While this descriptive study has established the feasibility of examining the effects of the lockdown on smartphone-derived step counts, it was based on aggregated data without providing any information regarding the smartphone users, nor did it examine individual characteristics associated with step count trajectories during the lockdown.

In the current study, we aimed to address several major limitations described above by using device-based, prospectively collected, continuous monitoring of daily step counts of a cohort of Chinese adults living in Shanghai spanning over 200 days across different phases of the COVID-19 pandemic. Our objectives are to determine: 1) the change in daily steps in response to the lockdown and reopening during the COVID-19 pandemic in China, and 2) the characteristics associated with trajectories in step counts during and after the lockdown. Findings from the study will not only help evaluate effects of COVID-19 restrictions on population-level physical activity, but also identify the subpopulations at risk of a sedentary lifestyle during the pandemic to inform preventive intervention strategies.

## Methods

### Sample and procedures

The study was conducted in Shanghai, China, an economically advanced city in East China with a population of more than 27 million people. We used step count data of a sample of 815 Chinese adults who participated in a 13-week step intervention which took place between September and November 2019. Participants were sampled from 11 workplaces in Pudong District, Shanghai. Participants were eligible if they were aged 18 years and above, without major chronic disease or disability, not pregnant, and without any intention to leave the current workplace in 12 months. Following the completion of the intervention on 30/11/2019, participants entered a nine-month follow-up phase which allows the research staff to access their step count data until 31/08/2020. Although the intervention group had higher step counts upon the completion of the intervention, by 20/12/19, the difference between groups had substantially reduced. Therefore, we consequentially used both the intervention and control groups as a cohort for continuous step tracking (Supplementary Fig. 1). All study procedures have been approved by the Shanghai Municipal Center for Disease Control and Prevention Ethical Review Committee (ChiCTR1900023813) and all participants in the study provided written informed consent before taking part in the study.

### Measures

#### Step counts

Daily step count was measured by WeRun, a social fitness plugin built in WeChat, the most popular mobile social media application in China. WeRun imports step count data from a smartphone’s built-in accelerometer, [18] and during the study participants shared their daily step counts via a cloud-based secure server. Based on a pilot study conducted prior to the intervention, step counts according to WeRun correlated strongly (spearman correlation coefficient 0.766, *p* < 0.001) with step counts measured by a hip-worn accelerometer (Actigraph GT3x-BT). We considered days with less than 1000 steps as an invalid wearing day (Supplementary Table 1) and daily step counts were truncated at 30,000 steps/day.

Baseline characteristics of participants were collected by trained research assistants. Participants reported demographic characteristics, including birth year and month, sex, marital status, educational attainment, and income, through a self-administered questionnaire. A trained interviewer was present during the completion of the questionnaire in case of questions and to ensure the completion of the questionnaire. Baseline physical activity was measured using the validated Chinese version of the International Physical Activity Questionnaire short form [19] and was categorised as sufficiently active (≥1000 metabolic equivalent [MET] minutes/week) vs insufficiently active (< 1000 MET minutes/week) [20]. We used the cut point that approximates the upper end of the current recommendations, [7] because the participants were overall highly active. Adiposity was measured using body mass index (BMI, calculated from measured height and weight (TCS-150 electric scale and Omron HBF-214 Body Composition Monitor Scale), categorised using Asian-specific cut-points as lower risk (< 23), increased risk (23–27.4), and high risk≥ (27.5) [21]) and body fat percentage using the reliable and valid [22] Omron HBF-214 Body Composition Monitor Scale (≤ 20 vs > 20 for men and ≤ 30 vs > 30 for women [23]).

### Timeline

Located more than 800 km East of Wuhan, Shanghai has been moderately affected by the pandemic. As of 31/08/2020, Shanghai recorded a total of 903 cases and 7 deaths. On 24/01/2020, following the Central Government’s Public Health Emergency Response Policies, Shanghai enforced ‘Level 1 (the highest level of) Restrictions’ with residents ordered to stay at home and all public places closed. The start of the COVID-19 Pandemic overlapped with the Chinese New Year, when most people were expected to gather with family and friends. Therefore, the initial two weeks of the lockdown was the most stringent. Between 10/02/2020 (the end of the Chinese New Year holiday) and 22/03/2020, some essential workers had returned to work while others continued to work from home for longer periods, and essential travels (e.g, grocery shopping) were allowed but controlled. Meanwhile, some public facilities and spaces gradually started to reopen. On 23/03/2020, Shanghai Government officially downgraded restrictions from Level 1 to Level 2. By then, most employees had returned to work and public spaces had started to reopen with control measures, such as extensive cleaning, temperature check, and crowding prevention. Acknowledging the gradual process of the reopening, we chose 22/03/2020 as ‘the end of the lockdown’ as this date represents an official down-grading of restrictions and returning to ‘normal life’. Throughout the paper, we refer to the period before 24/01/2020 as ‘before the lockdown’, the period between 24/01/2020 and 22/03/2020 as ‘during the lockdown’ and the period between 23/03/2020 and 08/07/2020 (last day of step data) as ‘after the lockdown’ or ‘reopening’.

### Statistical analysis

We linked step count data with baseline survey and biometric measures. For Research Aim 1 (change in step counts following the lockdown), we plotted the average daily step counts and the 95% confidence interval across the entire study period, for the entire sample and separately by the intervention and control groups. We used a ‘sharp’ regression discontinuity design (RDD) with cluster robust standard errors and binsize = 1 day to test the effect of the lockdown on participants’ step counts [24]. RDD is a causal inference method to evaluate whether an ‘abrupt’ change is causal by an intervention (i.e., the lockdown), by comparing the outcomes (i.e., step counts) following the intervention with the counterfactual without the intervention. Through minimising unobserved confounding, this quasi-experimental approach is designed to be analogous to a randomised control trial [24]. We used the Imbens and Kalyanaraman (IK-BW) method to determine the optimal bandwidth (i.e. number of days around either side of the lockdown date) for estimates of ‘local’ effect [25]. Based on the IK-BW method, we selected the bandwidth to be 26 days around the lockdown date (i.e., 13 days before and 13 days after). As a standard sensitivity analyses, we repeated the analysis using half (12 days, 6 days before and 6 days after) and double the bandwidth (50 days, 25 days before and 25 days after) around the lockdown date [25]. We conducted the RDD analysis for the start of the lockdown only, as the easing of lockdown was a more gradual process and there were no ‘abrupt’ changes in step counts.

For Research Aim 2 (determine characteristics associated with trajectories in step counts), we first built a linear mixed model with participants as a cluster variable, period (i.e., pre-lockdown, lockdown, and post-lockdown) and day as independent variables and step count as the dependent variable. We then tested a two-way interaction term (day x period) in the model to test whether step counts trajectories differed by period (i.e., comparing the lockdown and reopening periods with pre-lockdown as the reference). Finally, we tested whether baseline physical activity levels, adiposity (BMI and body fact percentage) and selected demographic characteristics (age: under 40 vs 40+ years, sex, marital status: married vs unmarried/divorced/widowed, education: university vs no university education) moderated the change in step count trajectory by testing a three-way interaction term (day x period x covariate of interest—e.g., baseline adiposity). All models were adjusted for relevant covariates (i.e., age, sex, education, income, marital status, worksite, intervention group allocation, baseline physical activity and baseline adiposity). We used R software for all our computations. The significance level was set at *p*-value 0.05, two-tailed. We provide an example of the codes used to run all the analysis in supplementary files.

## Results

Participant characteristics are summarised in Table 1. Averaged 40 years of age, nearly two thirds of the participants were female, more than 60% had a university degree or higher, and more than four in five were married. At baseline, slightly less than half of the participants were classified as normal weight, nearly 40% overweight and 13% obese based on BMI risk classification specific to Asian populations [21]. Based on body composition, more than two thirds were classified ‘at risk’. Participants had high levels of physical activity overall with two thirds reporting at least 1000 MET minutes of physical activity per week. Compared with a previous surveillance survey among a population-representative sample of working-age adults in Shanghai, [26] participants from the current study have higher education and reported higher levels of physical activity.

**Table 1:** Participants characteristics at baseline (*n* = 815)^a^

Variables	n (%)*
Age (year)	
20–29	117 (14.4)
30–39	309 (38.1)
40–49	259 (31.9)
50+	126 (15.5)
Sex	
Male	285 (35.0)
Female	530 (65.0)
Education	
High school graduate or below	161 (19.9)
Vocational training	154 (19.0)
University or higher	496 (61.2)
Marital status	
Married	672 (83.3)
Single/divorced/widowed	135 (16.7)
Body Mass Index (kg/m2)	
Low risk (< 23)	387 (47.8)
Increased risk (23–27.4)	318 (39.3)
High risk (≥27.5)	105 (13.0)
Body fat percentage	
Lower risk (≤20% for men and ≤ 30% for women)	263 (32.8)
Higher risk (> 20% for men and > 30% for women)	539 (67.2)
Baseline physical activity levels	
Insufficient (< 1000 MET^b^ minutes/week)	256 (33.2)
Sufficient (≥1000 MET^b^ minutes/week)	514 (66.8)

^**a**^Sample sizes for some variables deviated slightly from 815 due to missing data. Participants with missing data: age (*n* = 4), education (*n* = 4), martial status (*n* = 8), BMI (*n* = 5), body fat (*n* = 13), baseline physical activity (*n* = 45). ^b^MET = metabolic equivalent

**Fig. 1:**
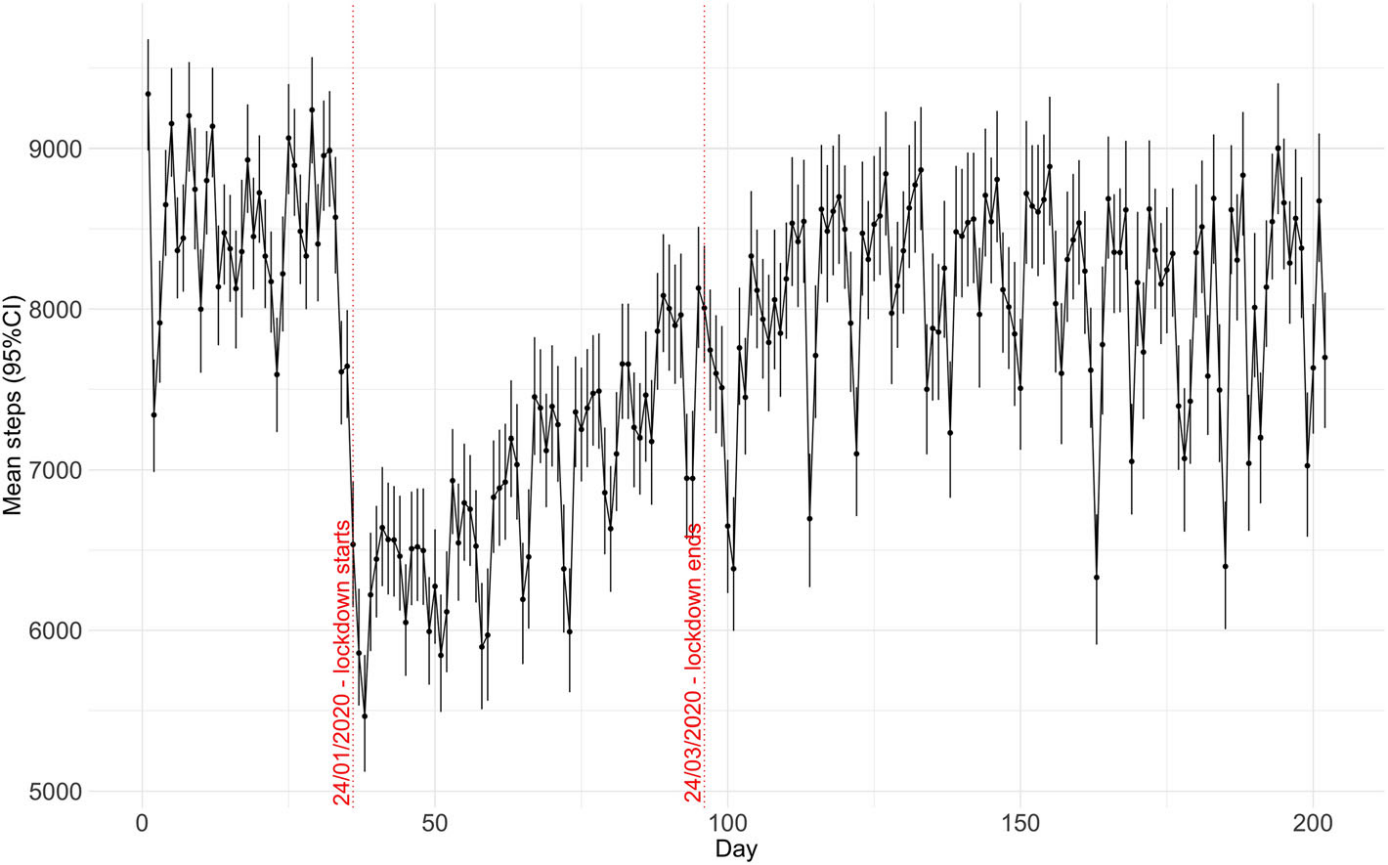
Mean daily steps (95% CI) of participants before, during and after the lockdown

**Fig. 2:**
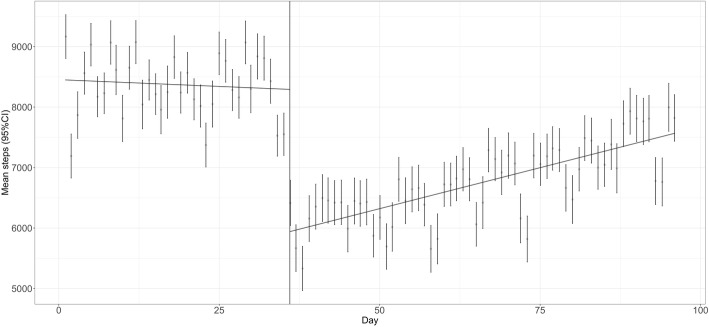
Graphic display of regression discontinuity analysis

Figure 1 showed average daily steps over 202 days for the entire sample (data for the intervention and control groups separately were presented in Supplementary Fig. 2). Overall, prior to the lockdown, the daily step counts averaged above 8000, which remained stable without statistically significant changes until the day before the lockdown (*p* = 0.073). On the first day of the lockdown (24/01/2020, Day 36 of the study period), the average daily step counts declined abruptly by 3796 steps (SE = 88). Results from RDD analysis (Fig. 2) suggested a sharp drop in step counts because of the lockdown (*p* < .001). Sensitivity analyses with half and double chosen bandwidths confirmed these results (Table 2). Since the initial decline, there was a significant trend in step count increase by 34 steps per day (ß = 34.1; SE = 2.5; *p* < .001). This increasing trend continued until the end of the lockdown (23/03/2020, Day 95) where the daily step counts recovered to only 708 steps (SE = 85) lower than on Day 1. Since then and until the end of our study period (09/07/2020, Day 202), daily step counts continued to increase by 5 steps per day (ß = 5.0; SE = 2.3; *p* = 0.029).

**Table 2:** Effects of the lockdown on step counts of participants (*n* = 815)

Bandwidth	Observations (person-day)	β (SE) (steps/day)	*p*-value
IK-bandwidth	32,424	− 2158 (126.20)	<.001
Half- bandwidth	15,973	− 1839 (139.38)	<.001
Double- bandwidth	54,374	− 2253 (120.87)	<.001

IK, Imbens and Kalyanaraman; SE, Standard Error

Models are adjusted for age, sex, education, income, marital status, intervention group allocation, baseline physical activity and body mass index

All models use robust standard errors with participant ID as a cluster unit

**Fig. 3:**
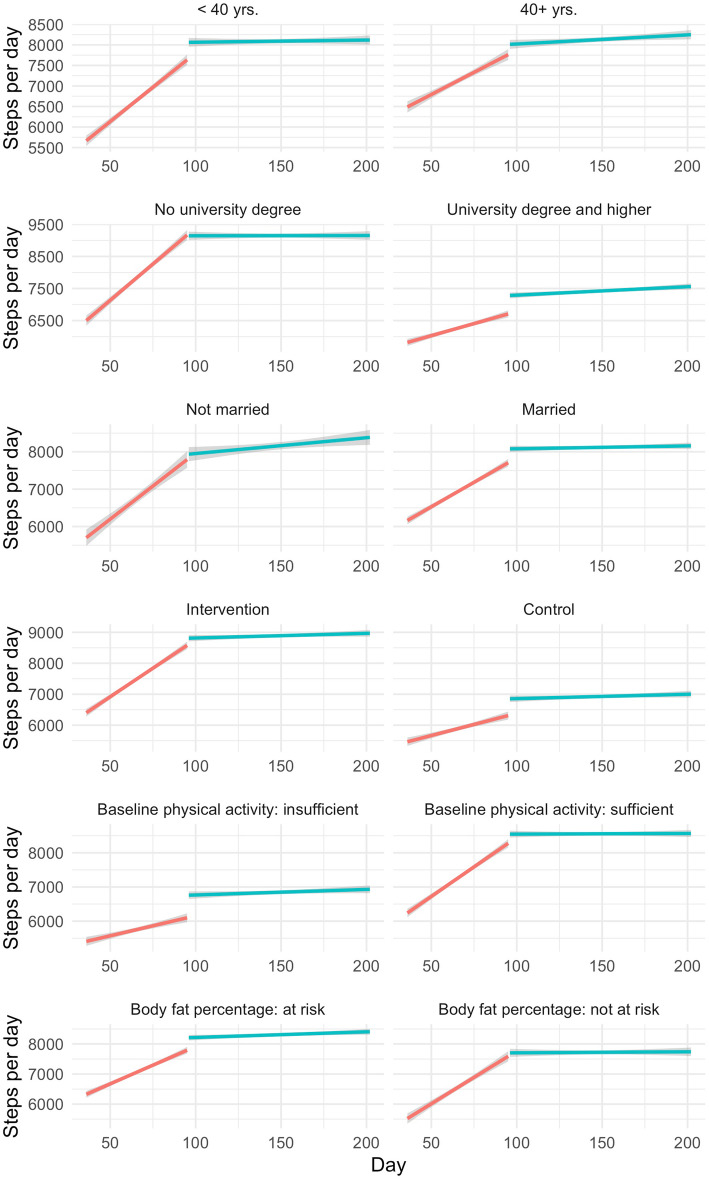
Unadjusted stratified trajectory of steps counts during and after the lockdown

We tested whether the trend in daily steps during and after the lockdown differed by baseline characteristics and found that age, education, marital status, intervention allocation, baseline physical activity and body composition significantly modified step count trajectories (Table 3). Specifically, the increase in steps during the lockdown was attenuated in those aged 40 years and above (ß = − 20.5; SE = 5.0; *p* < 0.001; interpreted as 20.5 fewer steps of increase per day compared with those aged below 40 years, those who were married (ß = − 22.6; SE = 6.9; *p* < 0.001), those with university education (ß = − 23.8; SE = 5.2; *p* < 0.001), those who were in the control group of the previous intervention (ß = − 24.9; SE = 5.2; *p* < 0.001), those who were insufficiently active at baseline (ß = − 27.5; SE = 5.3; *p* < 0.001), and those with a body composition classified as ‘at risk’ (ß = − 27.8; SE = 5.4; *p* < 0.001). During the reopening phase, the increase in step counts was again attenuated in those aged 40 years and above (ß = − 11.1; SE = 2.3; *p* = 0.02), those who were married (ß = − 15.9; SE = 6.3; *p* = 0.01), and those with ‘at risk’ body composition (ß = − 18.0; SE = 4.9; *p* < 0.001). However, after the lockdown restrictions were eased, those with university education had significantly larger increases in steps instead (ß = 11.9; SE = 4.8; *p* = 0.013), but there was no longer a difference in step count trajectories by baseline physical activity levels (ß = − 6.0; SE = 4.9; *p* = 0.22). We did not find significant effect modification by sex or BMI categories during or after the lockdown. For data visualisation, we stratified step count trajectories by significant effect modifiers during and after the lockdown (Fig. 3).

**Table 3:** Test of effect modification: differences in the trajectory of daily step counts before, during, and after the lockdown (*n* = 815)

	During lockdown		After lockdown	
	β (SE)^a^	*p*-value	β (SE) ^a^	*p*-value
Age (40+ vs 20–39 years)	−20.46 (5.02)	<.001	−11.07 (4.61)	0.016
Sex (female vs male)	−2.75 (5.29)	0.602	0.31 (4.86)	0.947
Martial status (married vs not married)	−22.62 (6.90)	0.001	−15.89 (6.33)	0.012
Education (university vs no university)	−23.80 (5.24)	<.001	11.88 (4.81)	0.013
Baseline physical activity (insufficient vs sufficient)	−27.52 (5.33)	<.001	−6.01 (4.89)	0.219
BMI (overweight/obese vs normal weight)	−8.81 (5.03)	0.079	−9.05 (4.62)	0.050
Body composition (at risk vs not at risk)	−27.82 (5.35)	<.001	−17.95 (4.91)	<.001
Intervention group allocation (control vs intervention)	−24.94 (5.17)	< 0.001	−3.69 (4.75)	0.437

^a^Interpreted as the differences in the change of daily step counts. For example: those aged 40+ years increased step counts by 20.46 fewer steps than those aged 20–39 years during the lockdown period

^b^Test of effect modification was conducted in a linear mixed model with participants as a cluster variable, period (i.e., pre-lockdown, lockdown, and post-lockdown) and day as independent variables and step count as the dependent variable. Effect modification was tested one at a time adjusted for age, sex, education, income, marital status, worksite, intervention group allocation, baseline physical activity and baseline adiposity

## Discussion

In this study, we used prospectively collected step count data over 202 days and 164,630 person-days to examine the effects of COVID-19 lockdown restrictions had on physical activity in Shanghai, China. To our knowledge, this is the first prospective study on the effects of COVID-19 lockdown and reopening on an objectively measured physical activity outcome. Our study revealed potential longer-term consequences of lock-down restrictions on daily step counts and identified specific population subgroups that may be particularly susceptible to declines in physcial activity, such as those who were older, married, insufficiently active at baseline and those with ‘at risk’ body composition.

Findings from our study extended evidence from previous survey-based studies of physical activity declines following the COVID-19 lockdown restrictions. For example, based on an online survey of 1491 adults in Australia, conducted in April 2020, 49% of the participants reported declined physical activity, while 21% reported increased physical activity [12]. Similarly, an online survey of 2381 Polish adults, conducted in April and May 2020, reported that 43% of participants decreased physical activity while only 19% increased [27]. Additionally, a Sport England survey conducted in April 2020 found that 41% of adults reported doing less physical activity while 31% reported doing more [28]. A Spanish study of 72 type 2 diabetes patients conducted in April and May 2020 found that participants’ weekly walking time declined from more than 300 min to just over 100 min [11]. These studies are subject to recall bias because data were collected retrospectively in response to the lockdown, by asking participants to compare activity levels before and after the lockdown. Such data could be informative and may be the only data that could be collected in most circumstances, considering the short notice usually given ahead of a lockdown and the amount of time required to obtain ethical approval and to design and conduct a study. In comparison, our study was opportunistic as COVID-19 happened during our ongoing data collection, therefore we could collect ‘true’ baseline data and follow up participants over time. Our findings, based on prospectively collected objective data, mostly confirmed findings from survey-based cross-sectional and retrospective studies.

Another advantage of our study is continuous data collection over 202 days, which provided a unique opportunity to examine the trajectory of step counts through different phases: before, during, and after the lockdown. Based on our data, there seemed to be a sharp initial drop in daily step counts by more than 40%, followed by slow but steady increase. This sharp decline may be a result of the abrupt nature of the lockdown. On 23/01/2020, the Chinese Government imposed a strict lockdown in Wuhan and banned all travels. By 24/01/2020, the local governments of 17 provinces (Shanghai included) implemented First-Level Public Health Emergency Response Policies. The date of the lockdown was particularly chosen to prevent social gatherings ahead of the Lunar New Year (starting 25/01/2020). In China, Lunar New Year is a public holiday when employees and students return home and it is a cultural tradition to visit families and friends and to gather for feasts and celebration. The Lunar New Year Holiday in 2020 was extended due to the pandemic. Therefore, the sharp decline in daily steps was a result of nearly everyone staying at home as a result of both the Lunar New Year and the lockdown. While step counts remained low for around two weeks, we observed slow but steady increase in step counts since then. This trajectory may be explained by the gradual process of returning to work. In Shanghai, the initial reopening of some workplaces and the resumption of the public transport system started on 10/02/2020. By 29/02/2020, nearly two thirds of all employees had returned to work. Although step counts continued to increase towards and beyond 23/03/2020 when the restrictions were officially eased, by the end of the study, 3.5 months into the reopening, the total step counts were still slightly below the baseline level. This finding may reflect people’s caution and hesitation that things have not returned 100% to normal.

According to our findings, lockdown restrictions affected some individuals’ step counts more than others. Previously, only a few studies examined the characteristics associated with retrospectively reported change in physical activity following the COVID-19 lockdown [12, 14–16]. Specifically, Reyes-Olavarria et al. found that those self-reported to be overweight were more prone to decreased physical activity [13]. We examined adiposity both in terms of measured BMI and body composition and found the latter to be a significant effect modifier both during and after the lockdown. Increasing evidence suggests that adiposity may be a predictor of physical activity declines [29, 30]. Our finding echoed this observation within the ‘unnatural’ context of lockdown restrictions. In terms of baseline physical activity, findings from our study suggest that in China, those who were more active at baseline recovered their daily step count more quickly after the initial decline. Such patterns continued beyond the reopening, implying that lockdown restrictions may disproportionately affect those who were less active. Furthermore, those in the intervention group, who started at a higher step count level than those in the control group, recovered their steps faster during, but not after, the lockdown. This difference may be explained by a sustained effect of the intervention, or the overall higher levels of physical activity among participants in the intervention group. Lesser et al. found that insufficiently active individuals were more likely to reduce physical activity than their active counterparts, [16] but Constandt et al. found the opposite - that those who were less active before COVID-19 were more likely to increase activities during the lockdown [14]. The role habitual physical activity plays in activity change during the lockdown may be context-specific. For example, outdoor exercise was permitted as ‘essential activity’ by some governments, such as those in Australia and the UK, but banned in Shanghai, China, between 24/01/2020 and 23/03/2020. Our recent analysis of Google query data suggested an unprecedented surge in online searches for exercise, particularly home-based exercise during the COVID-19 lockdown [31]. This suggests that lockdown restrictions may have prompted people to form new exercise habits, whether initiating exercise or adapting exercise routines according to restrictions. Interestingly, Constandt et al. found that 61% of those who were habitually inactive at baseline reported that there was more time to exercise during the lockdown, and those who were used to exercising with friends or in a club were more likely to reduce physical activity during the lockdown, despite being highly active at baseline [14]. This suggests that specific types of physical activity individuals were used to doing prior to the lockdown, their social context and adaptability to an indoor environment may determine ones’ ability to maintain physical activity during the lockdown.

Our study also identified some demographic characterises predictive of the trajectory in physical activity during and after the lockdown. For example, those who were older (40+ years) recovered step counts at a slower pace than their younger counterparts. This may be explained by the higher perceived susceptibility to COVID-19 and other competing priorities, such as home-schooling children, experienced in this age group. Furthermore, older Chinese adults typically exercise outdoors (e.g., Taichi, square dance) with social groups, [32] and may be more affected by lockdown restrictions than their younger counterparts. Our study also found that those who were married experienced a slower recovery in step counts than their unmarried counterparts. While this could also be a result of competing priorities, such as home-schooling or household chores, this pattern could also be an artefact of the lockdown policies. Between 24/01/2020 and 22/03/2020, the lockdown restrictions included reducing non-essential travel and limiting essential shopping trips to one per household per day in most communities. Therefore, unmarried adults and smaller households would receive more allowed opportunities for travel per person than married adults and larger households. Finally, the role education played was particularly interesting: while university-educated adults increased steps slower during the lockdown, they recovered steps faster after the lockdown. A possible explanation is that university-educated adults were more likely to be knowledge workers who had the privilege to work from home during the lockdown, and they may also have more financial resources to use private cars or taxis when they needed to travel. According to a recent Chinese national travel survey, during the COVID-19 pandemic, car and taxi trips accounted for 45% of all trips compared with 5.8% before the pandemic [33]. This change in modal share may have led to a reduction in incidental walking, particularly in those who can afford avoiding public transport.

### Strengths and limitations

Compared with other existing studies, our study has several important strengths, such as prospective data collection, objectively measured step counts, and continuous data of 164,630 person-days, spanning across 7 months before, during and after the lockdown. We also used innovative causal inference methods to confirm that the association between lockdown and step decline was causal. However, several limitations need to be acknowledged. First, we opportunistically used long-term follow-up data of a step count intervention. Although our baseline phase started after the between-group difference in step counts returned to null, we cannot be certain whether our baseline step counts were inflated as a result of the intervention. Second, we used smartphone-based accelerometers for measuring step counts, which can be subject to participants not wearing the phone consistently throughout the day. Particularly, smartphones may systematically underestimate home-based activities because participants are less likely to carry their phones continuously at home (as shown by data presented in Supplementary Table 1). Finally, because we opportunistically used a sample of intervention participants, which was not intended to be population-representative, findings from the current study have limited generalisability.

### Implications

Based on objectively measured step counts over 164,630 person-days, our study revealed a sharp decline in daily step counts immediately following the COVID-19 lockdown in Shanghai, China. Despite later increases in step counts during and after the lockdown, such a sustained period of lower physical activity could still have health implications, [34] such as metabolic derangements and change in body composition [35]. Considering such potential health consequences and the critical role physical activity plays in improving immune function, [36] governments and health professionals around the world should continue to encourage and facilitate physical activity during the pandemic. Other sectors, such as fitness/recreation and transportation, should use COVID-19 as an opportunity to upgrade service and management standard [37] and transform the current practice [38].

## Supplementary Information

**Additional file 1.**

## Data Availability

The datasets are available from the corresponding author on reasonable request.

## Abbreviations

COVID-19: Coronavirus disease-2019
WHO: World Health Organization
MET: metabolic equivalent
BMI: body mass index
RDD: regression discontinuity design
IK-BW: Imbens and Kalyanaraman
SE: standard error
