# Supplementary material for: How COVID-19 lockdown and reopening affected daily steps: evidence based on 164,630 person-days of prospectively collected data from Shanghai, China
Source: Int J Behav Nutr Phys Act. 2021 Mar 17;18:40. doi: 10.1186/s12966-021-01106-x (PMC7968558; doi:10.1186/s12966-021-01106-x)
Supplement: Supplementary file 1 — Additional file 1. [file 12966_2021_1106_MOESM1_ESM.pdf]

How COVID-19 lockdown and reopening affected daily steps: Evidence based on 164,630 person-days of prospectively collected data from Shanghai, China

Supplementary files

Supplementary Table 1. Average percentage of days with less than 1000 steps for participants (n=815)[a]

Variables	No. person-days with <1000 steps	Total No. person-days	% person-days with <1000 steps
Overall	20796	143834	7.37
Study phase			
Before the lockdown	471	28054	3.43
During the lockdown	3866	44219	13.24
After the lockdown	16459	71561	5.29
Age (year)			
20-29	3919	15473	8.26
30-39	9116	51888	7.68
40-49	5202	47116	6.91
50+	2383	28927	7.09
Missing	176	430	7.44
Sex			
Male	7643	49927	4.96
Female	13153	93907	8.66
Education			
High school graduate or below	3429	28891	5.79
Vocational training	4090	27018	8.16
University or higher	13261	87133	7.69
Missing	16	792	3.41
Marital status			
Married	15288	120254	7.22
Single/divorced/widowed	5088	22182	8.18
Missing	420	1398	7.65
Intervention allocation			
Intervention group	16008	87820	6.28
Control group	4788	56014	9.09
Baseline physical activity levels			
Insufficient (<1000 MET minutes/week)	6335	48205	8.41
Sufficient (≥1000 MET minutes/week)	14461	95629	6.85

Supplementary Figure 1. Participant Flow Diagram

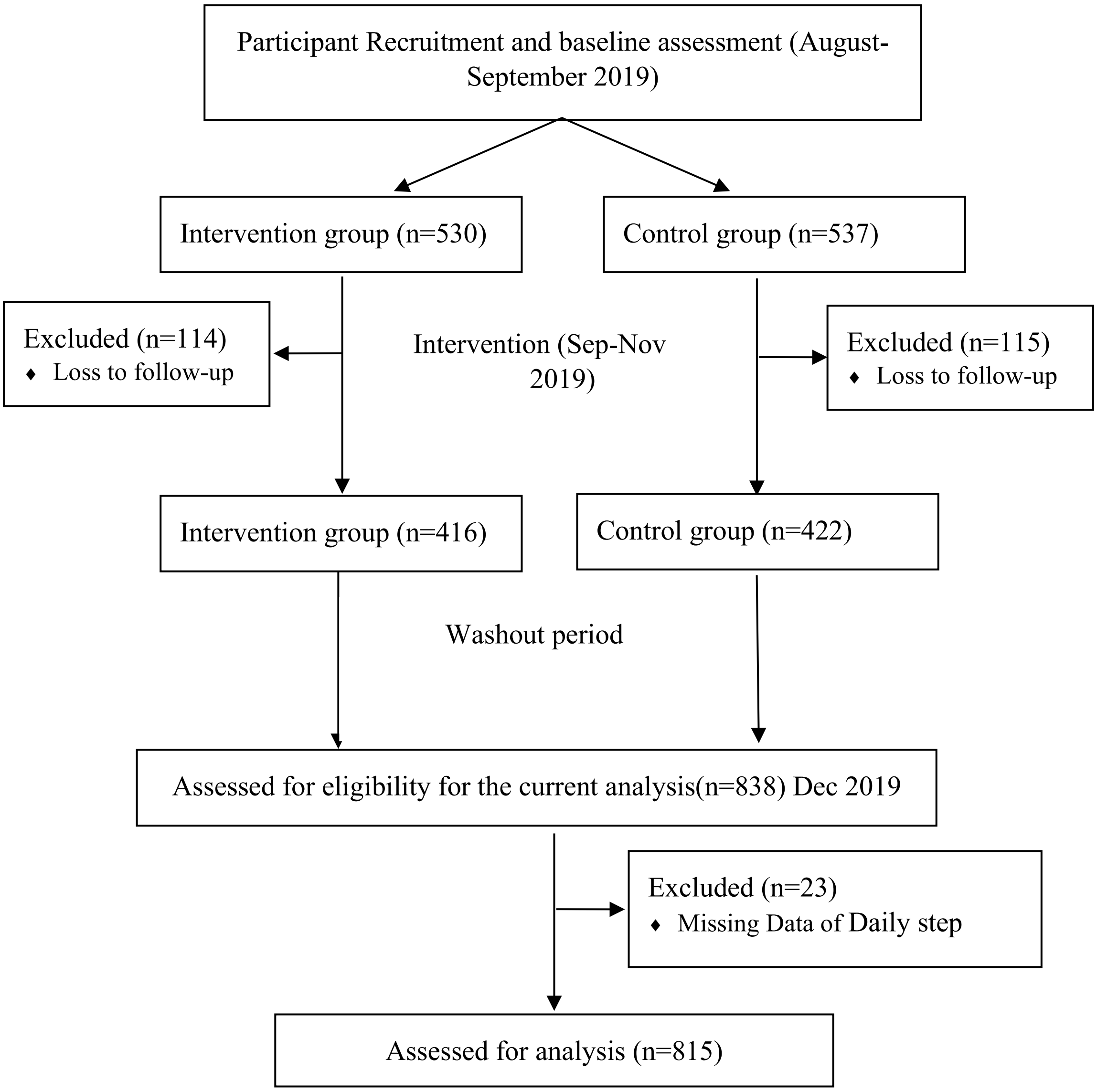

Supplementary Figure 2. Mean daily steps (95% CI) of participants in the intervention and control groups

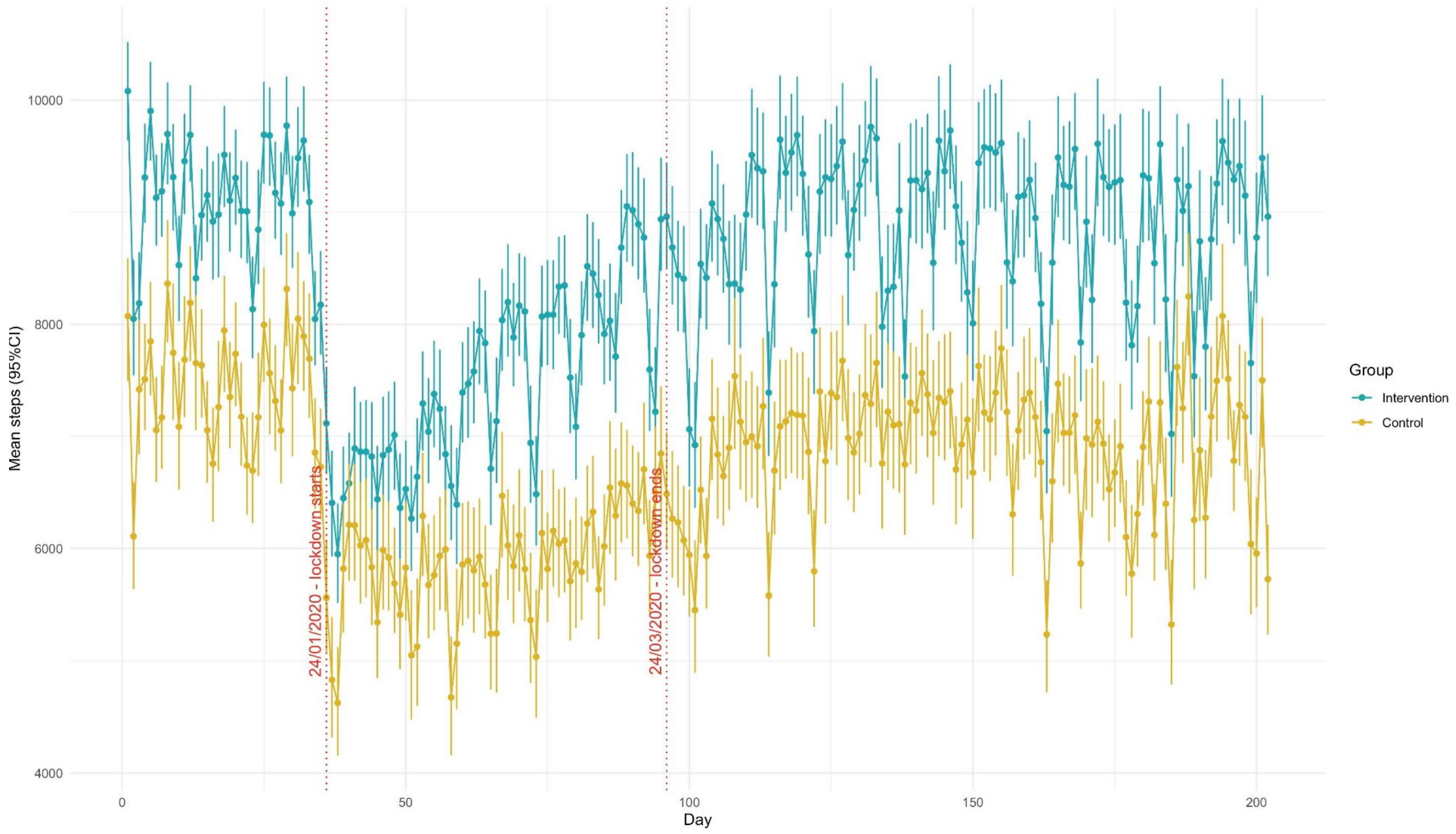

Supplementary file: R codes

```
#load required libraries
library(tidyverse)
library(hablar)
library(readstata13)
library(haven)
library(lme4)
library(lmerTest)
library(rdd)
 library(rdrobust)

#figure 1 day and dates
##all sample
tiff("/xx/steps_main.tiff", width = 14, height = 8, units = 'in', res = 300)
ggplot(daily, aes(x = visit, y = steps)) +
  stat_summary(fun.y = mean, geom = "point") +
  stat_summary(fun.y = mean, geom = "line") + #stat_smooth() +
  stat_summary(fun.data = mean_cl_boot, conf.int = .95, B = 5000, geom = "errorbar", width = 0.2) +
  labs(x = "Day", y = "Mean steps (95%CI)") +
  scale_color_manual(values = c("#00AFBB", "#E7B800")) +
  theme_minimal() +  geom_vline(xintercept = c(36,96),  linetype="dotted",
                         color = c("red", " red"), size=0.5) +
  annotate("text", label = "24/01/2020 - lockdown starts", x = 34, y =5800, size = 4, colour =
"red",angle=90) +
  annotate("text", label = "24/03/2020 - lockdown ends", x = 94, y = 5800, size = 4, colour =
"red",angle=90)
dev.off()

#RDD – we use a dataset called daily5
  covs = cbind.data.frame(daily5$bmi, daily5$uni_edu, daily5$married, daily5$worksite,
daily5$intervention, daily5$sex, daily5$income, daily5$age, daily5$met_minutes_week_calculated)
  tiff("/xx/rdd.tiff", width = 14, height = 8, units = 'in', res = 300)
  rdrobust::rdplot(daily5$steps,
               daily5$visit,
               c = 36,
               ci = 95, p=1, x.label = "Day", y.label = "Mean steps (95%CI)", title = "",
               covs = covs, covs_eval = "mean" , col.lines = "black", col.dots = "grey" )

  dev.off()
  #cluster robust standard errors – figures associated with plot
  lm_rdd_nonoutcome = rdd::RDestimate(steps ~ visit| bmi + uni_edu + married + worksite + group +
sex + income +  age+ met_minutes_week_calculated, daily5, cutpoint = 36, cluster = daily5$ID)
  summary(lm_rdd_nonoutcome)


#linear mixed model

model1<-  lmerTest::lmer(steps ~ visit + int + visit*int*bmi_cat2 + uni_edu + married + worksite +
group + sex + income +  age_cat+ pa_cat + (1|ID), data = daily)
summary(model1)
library(car)
```

```
coef_M <- fixef(model1)
vcov_M <- vcov(model1)
pnam <- names(coef_M)
dimnames(vcov_M) <- list(pnam,pnam)
print(deltaMethod(model1, "visit + `visit:int2:age_cat40+ yrs` ",
              vcov. = vcov_M, parameterNames = pnam))
"visit + `visit:int1` "


####separated plots####
#BF, PA, AGE, EDUCATION, MARITAL STATUS
#BF
p5<-ggplot(daily[!is.na(daily$BF_cat),], aes(x = visit, y = steps,group= int_cat, color = int_cat))
+stat_smooth(formula = y ~ x) +
  theme_minimal()  +  labs(x = "Day", y = "Steps per day")+
  scale_colour_discrete("Period around lockdown") + facet_wrap(~BF_cat ) +xlim(36,202)+
  theme(legend.position = c(0.8, -10),
       legend.direction = "horizontal")
#PA
p4<-ggplot(daily[!is.na(daily$pa_cat),], aes(x = visit, y = steps,group= int_cat, color = int_cat))
+stat_smooth(formula = y ~ x) +
  theme_minimal()  +  labs(x = "Day", y = "Steps per day")+
  scale_colour_discrete("Period around lockdown") + facet_wrap(~pa_cat ) +xlim(36,202)+
  theme(legend.position = c(0.8, -10),
       legend.direction = "horizontal")
#AGE
p1<-ggplot(daily[!is.na(daily$age_cat),], aes(x = visit, y = steps,group= int_cat, color = int_cat))
+stat_smooth(formula = y ~ x) +
  theme_minimal()  +  labs(x = "Day", y = "Steps per day")+
  scale_colour_discrete("Period around lockdown") + facet_wrap(~age_cat ) +xlim(36,202)+
  theme(legend.position = c(0.8, -10),
       legend.direction = "horizontal")
#EDUCATION
p2<-ggplot(daily[!is.na(daily$uni_edu),], aes(x = visit, y = steps,group= int_cat, color = int_cat))
+stat_smooth(formula = y ~ x) +
  theme_minimal()  +  labs(x = "Day", y = "Steps per day")+
  scale_colour_discrete("Period around lockdown") + facet_wrap(~uni_edu ) +xlim(36,202)+
  theme(legend.position = c(0.8, -10),
      legend.direction = "horizontal")

#MARITAL
p3<-ggplot(daily[!is.na(daily$married),], aes(x = visit, y = steps,group= int_cat, color = int_cat))
+stat_smooth(formula = y ~ x) +
  theme_minimal()  +  labs(x = "Day", y = "Steps per day")+
  scale_colour_discrete("Period around lockdown") + facet_wrap(~married ) +xlim(36,202) +
 theme(legend.position = "bottom",
        legend.direction = "horizontal")

#ggarrange(p1,p2,p3,p4,p5, nrow = 5, ncol = 1)
tiff("/xx/moderators.tiff", width = 6, height = 10, units = 'in', res = 300)
ggarrange(p1 +
```

```
    theme(#axis.text.y = element_blank(),
          #axis.ticks.y = element_blank(),
          #axis.title.y = element_blank(),
          axis.title.x = element_blank()),
  p2 +
    theme(#axis.text.y = element_blank(),
          #axis.ticks.y = element_blank(),
          #axis.title.y = element_blank(),
          axis.title.x = element_blank()),

  p3 +
    theme(#axis.text.y = element_blank(),
          #axis.ticks.y = element_blank(),
          # axis.title.y = element_blank(),
          axis.title.x = element_blank()),
  p4 +
    theme(#axis.text.y = element_blank(),
          #axis.ticks.y = element_blank(),
         #axis.title.y = element_blank(),
          axis.title.x = element_blank()),
  p5 ,


  nrow = 5)

dev.off()
```

STROBE Statement—checklist of items that should be included in reports of observational studies

	Item No	Recommendation
**Title and abstract**	1	(*a*) Indicate the study's design with a commonly used term in the title or the abstract We specified 'perspective' in the title
		(*b*) Provide in the abstract an informative and balanced summary of what was done and what was found We provided an informative and balanced summary in abstract (P2)
**Introduction**		
Background/rationale	2	Explain the scientific background and rationale for the investigation being reported (P3-4)
Objectives	3	State specific objectives, including any prespecified hypotheses (P4, last paragraph of the introduction)
**Methods**		
Study design	4	Present key elements of study design early in the paper (P4, Sample and procedures)
Setting	5	Describe the setting, locations, and relevant dates, including periods of recruitment, exposure, follow-up, and data collection (P4, Sample and procedures)
Participants	6	(*a*) *Cohort study*—Give the eligibility criteria, and the sources and methods of selection of participants. Describe methods of follow-up (P4, Sample and procedures) *Case-control study*—Give the eligibility criteria, and the sources and methods of case ascertainment and control selection. Give the rationale for the choice of cases and controls *Cross-sectional study*—Give the eligibility criteria, and the sources and methods of selection of participants
		(*b*) *Cohort study*—For matched studies, give matching criteria and number of exposed and unexposed *Case-control study*—For matched studies, give matching criteria and the number of controls per case
Variables	7	Clearly define all outcomes, exposures, predictors, potential confounders, and effect modifiers. Give diagnostic criteria, if applicable (P5, measures)
Data sources/ measurement	8*	For each variable of interest, give sources of data and details of methods of assessment (measurement). Describe comparability of assessment methods if there is more than one group (P5, measures)
Bias	9	Describe any efforts to address potential sources of bias (P6-7, Statistical analysis: we used causal inference methods with multiple sensitivity analysis)
Study size	10	Explain how the study size was arrived at (Supplementary Figure 1)
Quantitative variables	11	Explain how quantitative variables were handled in the analyses. If applicable, describe which groupings were chosen and why (P5-7, measures and statistical analysis)
Statistical methods	12	(*a*) Describe all statistical methods, including those used to control for confounding (P6-7)
		(*b*) Describe any methods used to examine subgroups and interactions (P7)
		(*c*) Explain how missing data were addressed (All data points considered in the analysis)
		(*d*) *Cohort study*—If applicable, explain how loss to follow-up was addressed (outcomes were collected through mobile phone, therefore no loss to follow-up) *Case-control study*—If applicable, explain how matching of cases and controls was addressed *Cross-sectional study*—If applicable, describe analytical methods taking account of sampling strategy
		(*e*) Describe any sensitivity analyses (Statistical analysis, Table 2)

Continued on next page

**Results**		
Participants	13*	(a) Report numbers of individuals at each stage of study—eg numbers potentially eligible, examined for eligibility, confirmed eligible, included in the study, completing follow-up, and analysed (Supplementary Figure 1)
		(b) Give reasons for non-participation at each stage (Supplementary Figure 1)
		(c) Consider use of a flow diagram (Supplementary Figure 1)
Descriptive data	14*	(a) Give characteristics of study participants (eg demographic, clinical, social) and information on exposures and potential confounders (Table 1)
		(b) Indicate number of participants with missing data for each variable of interest (Table 1)
		(c) *Cohort study*—Summarise follow-up time (eg, average and total amount) (P7, 202 days)
Outcome data	15*	*Cohort study*—Report numbers of outcome events or summary measures over time (P7-8)
		Case-control study—Report numbers in each exposure category, or summary measures of exposure
		Cross-sectional study—Report numbers of outcome events or summary measures
Main results	16	(*a*) Give unadjusted estimates and, if applicable, confounder-adjusted estimates and their precision (eg, 95% confidence interval). Make clear which confounders were adjusted for and why they were included (P7-8. Tables 2 and 3)
		(*b*) Report category boundaries when continuous variables were categorized (N/A)
		(*c*) If relevant, consider translating estimates of relative risk into absolute risk for a meaningful time period (N/A)
Other analyses	17	Report other analyses done—eg analyses of subgroups and interactions, and sensitivity analyses (Sensitivity analysis Table 2, Interaction Table 3, Subgroup analysis Figure 3)
**Discussion**		
Key results	18	Summarise key results with reference to study objectives (P8-9)
Limitations	19	Discuss limitations of the study, taking into account sources of potential bias or imprecision. Discuss both direction and magnitude of any potential bias (P12, strengths and limitations)
Interpretation	20	Give a cautious overall interpretation of results considering objectives, limitations, multiplicity of analyses, results from similar studies, and other relevant evidence (P9-12)
Generalisability	21	Discuss the generalisability (external validity) of the study results (P12, strengths and limitations)
**Other information**		
Funding	22	Give the source of funding and the role of the funders for the present study and, if applicable, for the original study on which the present article is based (P13)

*Give information separately for cases and controls in case-control studies and, if applicable, for exposed and unexposed groups in cohort and cross-sectional studies.

Note: An Explanation and Elaboration article discusses each checklist item and gives methodological background and published examples of transparent reporting. The STROBE checklist is best used in conjunction with this article (freely available on the Web sites of PLoS Medicine at http://www.plosmedicine.org/, Annals of Internal Medicine at http://www.annals.org/, and Epidemiology at http://www.epidem.com/). Information on the STROBE Initiative is available at www.strobe-statement.org.
